# Efficacy and Safety of Dulaglutide for Weight Reduction Among Diabetic Patients in Saudi Arabia: A Retrospective Cohort Study

**DOI:** 10.7759/cureus.88424

**Published:** 2025-07-21

**Authors:** Gadah K Alonazi, Lamia A Alawuad

**Affiliations:** 1 Pharmaceutical Care Department, Prince Sattam bin Abdulaziz University Hospital, Al-Kharj, SAU; 2 Pharmaceutical Care Department, Prince Mohammed bin Abdulaziz Hospital, Al-Riyadh, SAU

**Keywords:** dulaglutide, glp-1 receptor agonist, glycemic control, type 2 diabetes, weight loss

## Abstract

Background and objective

Obesity commonly affects individuals with type 2 diabetes mellitus (T2DM) and can lead to serious health complications. Glucagon-like peptide-1 receptor agonists (GLP-1 RAs) are being explored for their potential role in promoting weight loss and improving glycemic control. However, data on its effectiveness in real-world settings, particularly in Saudi Arabia, remains limited. The objective of this study was to evaluate the efficacy and safety of dulaglutide in weight reduction and glycemic control among diabetic patients in Saudi Arabia.

Methods

A retrospective cohort study was conducted at Prince Mohammed bin Abdulaziz Hospital in Riyadh. A total of 132 adult patients who were diagnosed with T2DM and received dulaglutide (1.5 mg/week) between January 1, 2019, and December 1, 2021, were included. Changes in body mass index (BMI) and hemoglobin A1c (HbA1c) were assessed at baseline, six months, and 12 months. Safety outcomes, including side effects like hypoglycemic events, were recorded. Paired t-tests and descriptive statistics were performed using IBM SPSS Statistics version 26.0 (Armonk, NY: IBM Corp.) for Windows.

Results

A significant reduction in BMI was observed at six months (mean decrease from 36.96±6.51 to 36.23±6.78 kg/m², p=0.023), while changes at 12 months were not statistically significant (p=0.178). HbA1c levels decreased significantly over 12 months (from 9.69±1.78% to 8.31±1.62%, p=0.001), (statistical significance was defined as a p<0.05). A total of 24.2% of the patients had hypoglycemia, predominantly among insulin users; adverse effects were reported by 10.6% of patients, such as constipation, nausea, and diarrhea.

Conclusion

Dulaglutide demonstrated efficacy in weight reduction and significant long-term improvement in glycemic control among Saudi Arabian patients with T2DM. It was well tolerated, with a low incidence of adverse effects. These findings support the role of dulaglutide as a therapeutic option for weight and glucose management in diabetic populations.

## Introduction

Obesity is the excessive or abnormal accumulation of fat or adipose tissue in the body [1]. The World Health Organization (WHO) defines overweight as a body mass index (BMI) ≥25 kg/m^2^ and obesity as a BMI ≥30 kg/m^2^ [2]. Obesity is associated with higher rates of death driven by comorbidities, such as type 2 diabetes mellitus, dyslipidemia, hypertension, obstructive sleep apnea (OSA), and certain types of cancer [3]. According to the World Health Organization (WHO), at least 2.8 million people die annually because of being overweight or obese [2]. The global burden of obesity is estimated to increase dramatically according to a recent estimate. By 2030, the number of overweight and obese adults is projected to reach 1.35 billion and 573 million, respectively [4]. In Saudi Arabia, the local burden is similarly concerning. A recent national survey conducted in 2020 reported an obesity prevalence of 24.7% among the Saudi population [5]. Obesity is a major risk factor for cardiovascular diseases, diabetes, musculoskeletal disorders, and cancers [6]. Recently, obesity has led to the progression of major complications of COVID-19 [7].

The treatment of obesity can improve quality of life and help patients reduce the risk of obesity-related complications like hypertension and T2DM. The management of obesity includes nutrition, physical activity, pharmacological treatment, and surgery. The pharmacological treatment is recommended for patients with a BMI ≥30 kg/m^2^ or a BMI ≥27 kg/m^2^ with an obesity-related disease (e.g., hypertension, type 2 diabetes mellitus, sleep apnea) [8]. Currently approved pharmacological agents for weight management include liraglutide, semaglutide, and orlistat; these agents act through different mechanisms, such as appetite suppression, nutrient absorption inhibition, or enhancement of satiety, and are considered based on individual patient profiles, tolerability, and comorbidities [8].

Glucagon-like peptide 1 receptor agonists (GLP-1 RAs) are incretin hormones used to treat adult patients with type 2 diabetes mellitus. According to the American Diabetes Association (ADA), metformin remains the preferred first-line therapy for treating T2DM. The addition of a GLP-1 receptor agonist should be considered in patients who are intolerant to or have contraindications for metformin, as well as in those whose hemoglobin A1c remains more than 1.5% above target or who fail to achieve glycemic goals within three months, particularly in individuals with established atherosclerotic cardiovascular disease, heart failure, or chronic kidney disease [9]. In addition to their glucose-lowering and cardioprotective effects, recent studies have demonstrated that GLP-1 receptor agonists also provide clinically meaningful benefits in weight reduction, making them a valuable option for patients with type 2 diabetes and coexisting obesity [10]. GLP-1 agonists enhance glucose-dependent insulin secretion by the pancreatic beta cell, suppress inappropriately elevated glucagon secretion, slow gastric emptying, decrease appetite, and increase satiety by direct actions on the hypothalamus [11]. In February 2017, Htike conducted a systematic review evaluating the efficacy and safety of glucagon-like peptide-1 receptor agonists (GLP-1 RAs) in patients with type 2 diabetes [11]. The review included 34 clinical trials with a total of 14,464 participants. The results demonstrated that all GLP-1 RAs significantly reduced glycated hemoglobin (HbA1c), while all except albiglutide also led to weight reduction [12], and in double-blind placebo-controlled trial conducted in March 2021, in 16 countries, to study the effect of once weekly semaglutide in 1961 overweight or obese adults, the results showed the mean change in body weight from baseline to week 68 was -14.9% in the semaglutide group as compared with -2.4% with placebo [13]. In addition, the treatment with GLP-1 RA has significantly reduced cardiovascular disease risk and mortality and improved renal outcomes [14]. Evidence suggests that the physiological effects of GLP‑1 are diminished in individuals with obesity and type 2 diabetes. This reduced GLP‑1 activity may help explain certain metabolic abnormalities observed in obesity, such as accelerated gastric emptying and weakened satiety signaling. As a result, dysfunction in incretin signaling could represent a key pathophysiological link between obesity and type 2 diabetes [15]. Thus, the GLP-1 RAs have proven to be effective for obesity treatment in addition to glycemic control.

Dulaglutide is one of the long-acting GLP-1 RAs administered as a once-weekly subcutaneous injection approved for the treatment of adults with T2DM in 2014, and it can be used as a monotherapy or in addition to an oral hypoglycemic or insulin therapy [16]. Although dulaglutide is not approved for obesity treatment, it caused a significant reduction in weight in several clinical studies. We need more studies to measure dulaglutide's effectiveness in weight reduction.

The rising rates of obesity in Saudi Arabia are a significant issue, as obesity is linked to various health risks. To date, there is limited evidence on the efficacy and safety of dulaglutide for weight loss in Saudi Arabia. This study aimed to assess the efficacy and safety of dulaglutide for weight reduction in diabetic Saudi patients.

## Materials and methods

Study design and setting

This retrospective cohort study was conducted at Prince Mohammed bin Abdulaziz Hospital, a tertiary care facility located in Riyadh, Saudi Arabia. The study period extended from January 1, 2019, to December 1, 2021. Ethical approval was obtained from the IRB Committee at King Fahad Medical City, Riyadh (#22-030E).

Patient selection

Adult patients aged 18 years and older with a confirmed diagnosis of type 2 diabetes mellitus (T2DM) who were treated with dulaglutide (1.5 mg once weekly) for at least three months were included in this study; adherence was assessed by reviewing electronic refill records and physician documentation for any missed or delayed doses. Additional inclusion criteria included a body mass index (BMI) greater than 25 kg/m². Patients were excluded if they had type 1 diabetes, were pregnant, or had gestational diabetes.

**Figure 1:**
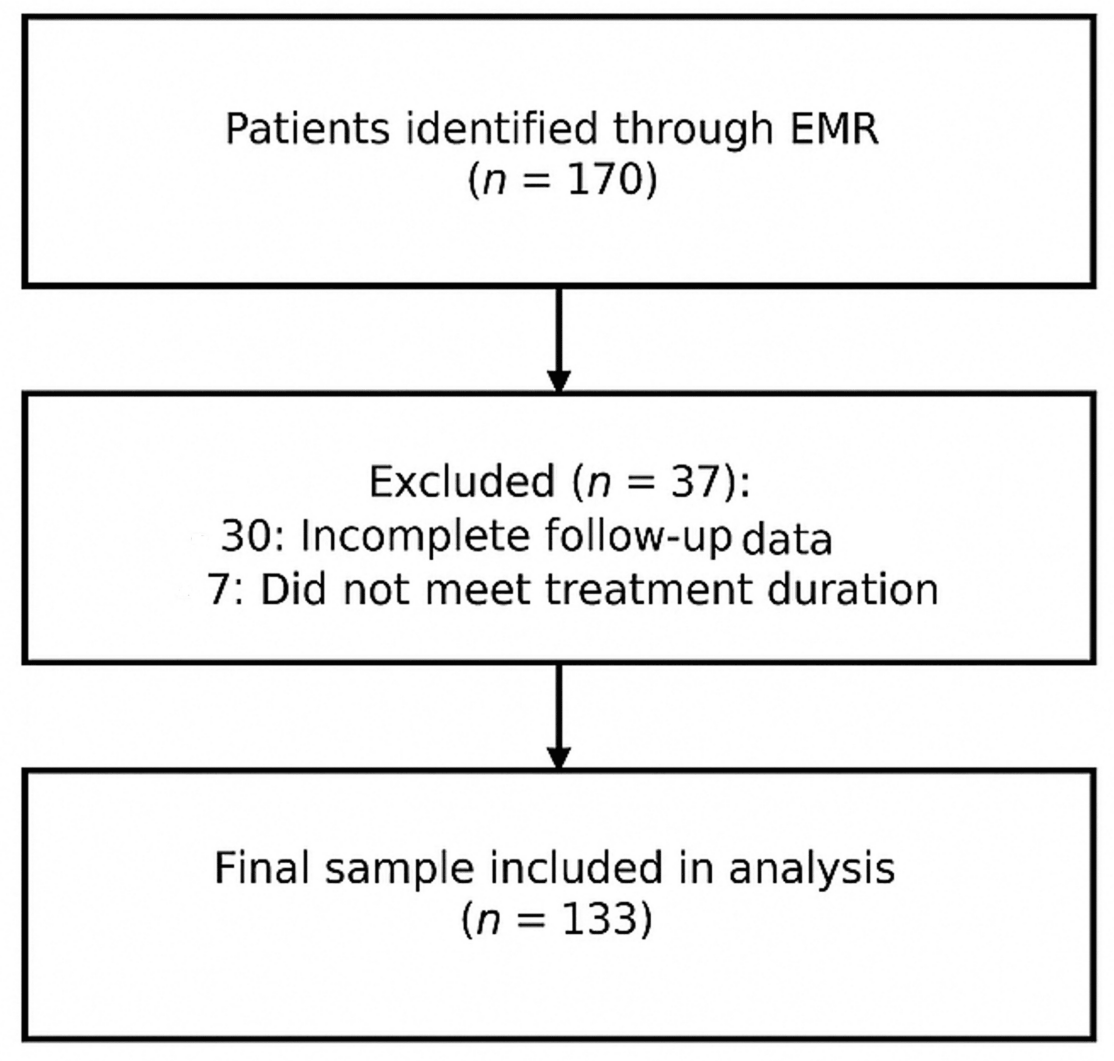
Patient flow diagram. EMR: electronic medical records

A total of 170 patients were initially identified through electronic medical records. Of these, 133 patients met the eligibility criteria and were included in the final analysis. The remaining patients were excluded due to incomplete follow-up data, insufficient treatment duration, or failure to meet inclusion criteria. The screening, inclusion, follow-up, and exclusion process is illustrated in Figure 1.

Data collection

Patient data were extracted from the hospital’s electronic health record system using a standardized collection form. The collected data included demographic information, such as age and gender; clinical data comprising baseline and follow-up measurements of body mass index (BMI) and hemoglobin A1c (HbA1c); and medication history detailing the use of dulaglutide and other antidiabetic agents, including insulin, metformin, sulfonylureas, SGLT2 inhibitors, and DPP-4 inhibitors. Lifestyle data captured the presence or absence of documented diet or exercise interventions, while safety data encompassed reported adverse effects, including gastrointestinal symptoms and hypoglycemic episodes. Follow-up data were gathered at baseline, six months, and 12 months after the initiation of dulaglutide therapy.

Outcomes

The primary outcome was the change in BMI over the study period. Secondary outcomes included changes in HbA1c and the incidence of adverse effects, specifically, a reduction in BMI of ≥5% and a decrease in HbA1c of ≥0.5% are recognized as clinically meaningful in the management of T2DM.

Statistical analysis

Data analysis was performed using IBM SPSS Statistics version 26.0 (Armonk, NY: IBM Corp.) for Windows. Descriptive statistics were used to summarize the study population. Continuous variables were presented as means and standard deviations, while categorical variables were expressed as frequencies and percentages. Paired sample t-tests were used to compare baseline and follow-up values for BMI and HbA1c. Independent t-tests and chi-square tests were used for subgroup comparisons. A p-value of less than 0.05 was considered statistically significant.

## Results

At baseline, 133 patients receiving a weekly dose of dulaglutide (1.5 mg) were enrolled in this study. As shown in Table 1, the mean age of the study population was 53.32±9.89 years (range: 25-73 years), and 65% of participants were female. Obesity was highly prevalent as follows: over 90% of patients had a BMI ≥30 kg/m², with 34% classified as class II obese, 29% as class III obese, and 8% as overweight.

**Table 1:** Baseline sample characteristics. HbA1c: hemoglobin A1c

Variables	n	%
Gender		
Male	46	35%
Female	87	65%
BMI		
Normal weight (BMI 18.5 to <25)	1	1%
Overweight (BMI 25 to <30)	11	8%
Obesity class I, II, III (BMI >30)	121	91%
HbA1c		
Normal (<5.7%)	1	1%
Pre-diabetes (5.7-6.4%)	2	1%
Controlled diabetes (6.5-8.0%)	21	16%
Uncontrolled diabetes (>8.0%)	109	82%
Patient was on diet or exercise		
Yes	32	24%
No	96	72%
Not available information	5	4%
Other antidiabetic medications		
Insulin	97	73%
Metformin	74	56%
Gliclazide	33	25%
Empagliflozin	10	8%
Sitagliptin	2	2%
Others	<1%	<1%
Dose of insulin		
>0.4 unit/kg/day	97	73%
<0.4 unit/kg/day	36	27%
Hypoglycemia event		
Yes	32	24%
No	101	76%

Regarding glycemic control, the mean baseline hemoglobin A1c (HbA1c) was 9.69±1.78%, indicating poor glycemic status at initiation. In terms of lifestyle management, 24% of patients reported adherence to a diet or exercise regimen, while 72% did not, and data were missing for 4% of participants.

A majority of patients (73%) were on insulin therapy, with most receiving high doses, specifically 73% of patients were on an insulin dose of more than 0.4 units/kg/day. Other commonly prescribed antidiabetic agents included metformin (56%), gliclazide (25%), empagliflozin (8%), and sitagliptin (2%). The frequent use of combination therapy reflects the complexity of managing poorly controlled type 2 diabetes mellitus (T2DM) in this population. Hypoglycemia was reported in 24% of patients during the treatment period, likely due to the concurrent use of insulin or sulfonylureas with dulaglutide.

Weight reduction outcomes

**Figure 2:**
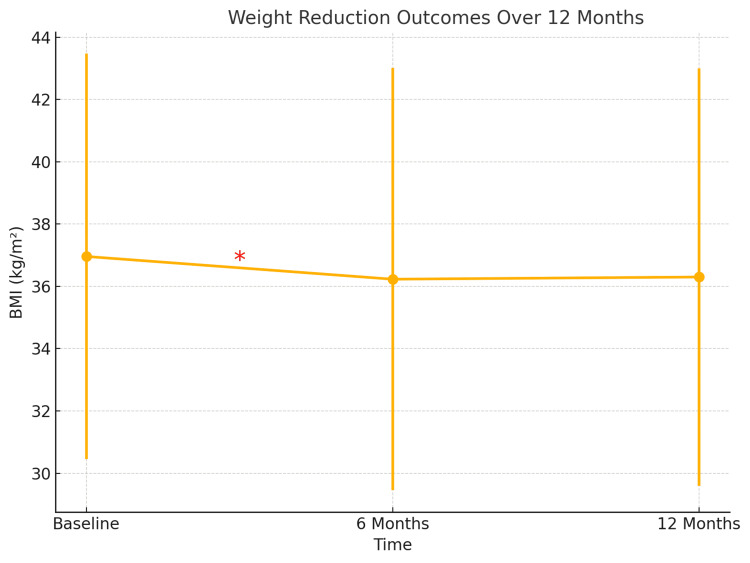
BMI change over time. *The asterisk symbol between baseline and six months indicates a statistically significant reduction in BMI (p=0.023).

Table 2 presents the results of paired sample t-tests comparing BMI at different time points. There was a statistically significant reduction in BMI from baseline (36.96±6.51 kg/m²) to six months (36.23±6.78 kg/m²; p=0.023), representing a modest absolute change of approximately 2.4%. However, the reduction from six to 12 months was not significant (p=0.818), and the overall change from baseline to 12 months was also not statistically significant (p=0.178). Figure 2 illustrates the trend in BMI over a 12-month period.

**Table 2:** BMI change over time.

Time points	Mean BMI (kg/m²)	Standard deviation	Compared to	p-Value
Baseline	36.96	6.51	-	-
6 Months	36.23	6.78	Baseline	0.023
12 Months	36.09	9.43	6 Months	0.818
	-	-	Baseline	0.178

Glycemic control outcomes

**Figure 3:**
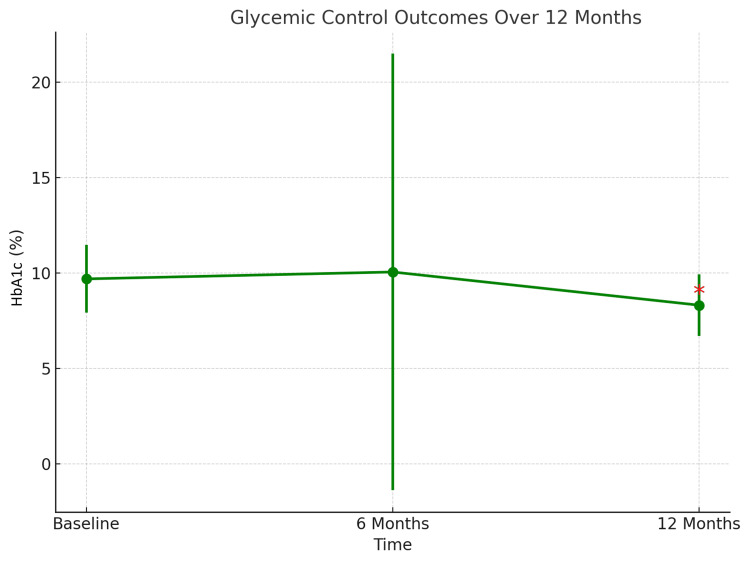
HbA1c change over time. The asterisk symbol indicates a statistically significant reduction in HbA1c between baseline and 12 months (p=0.001).

For the secondary objectives of this study, as summarized in Table 3, the mean HbA1c at baseline was 9.69±1.78%. After six months of dulaglutide use, HbA1c increased slightly to 10.05±11.44%, which was not significant (p=0.722). At 12 months, HbA1c significantly decreased to 8.31±1.62% (p=0.001 when compared to baseline). As shown in Figure 3, this suggests improved glycemic control throughout the treatment course.

**Table 3:** HbA1c change over time.

Time point	Mean HbA1c (%)	Standard deviation	Compared to	p-Value
Baseline	9.69	1.78	-	-
6 Months	10.05	11.44	Baseline	0.722
12 Months	8.31	1.62	Baseline	0.001

Safety profile

Regarding safety, summarized in Table 4, 11% of patients experienced gastrointestinal side effects, including constipation (2%), nausea (2%), vomiting (3%), diarrhea (1%), and abdominal pain (3%). A smaller portion (1%) reported a decrease in appetite. Hypoglycemia was reported by 32 patients (24%). The majority of patients who experienced hypoglycemia were also receiving insulin at doses exceeding 0.4 units/kg/day and sulfonylureas, which likely contributed to the incidence of hypoglycemia observed during the study, while 63 patients (47%) did not experience any adverse effects. Notably, the side effect profile was unknown for 22 patients (17%). Dulaglutide was well tolerated by the majority of participants, with nearly half of the patients reporting no adverse events.

**Table 4:** Reported side effects of dulaglutide.

Side effect	n	%
Constipation	3	2%
Diarrhea	1	1%
Nausea	3	2%
Vomiting	4	3%
Abdominal pain	4	3%
Decrease in appetite	1	1%
Hypoglycemia	32	24%
No side effect	63	47%
Unknown	22	17%

## Discussion

The present study investigated the efficacy and safety of dulaglutide, a GLP-1 receptor agonist, in reducing body weight and improving glycemic control in a sample of 133 Saudi patients with type 2 diabetes mellitus (T2DM). Over a follow-up period of up to 12 months, dulaglutide showed modest short-term weight loss and meaningful glycemic improvement.

In terms of weight reduction, a statistically significant decrease in BMI was observed six months of treatment (p=0.023), which is consistent with findings from previous clinical trials and real-world studies demonstrating the weight-lowering efficacy of GLP-1 receptor agonists [17,18]. However, the BMI reduction from six to 12 months did not maintain statistical significance. This may be attributed to several factors, such as medication adherence, lack of lifestyle intervention (e.g., physical activity, dietary compliance), or biological adaptation.

Although dulaglutide is not primarily indicated for weight management, the observed effect in the first few months supports its potential role in obesity management in diabetic patients. These findings support earlier research indicating that GLP-1 receptor agonists may assist in weight reduction through delayed gastric emptying, increased satiety, and appetite suppression [19].

Regarding glycemic control, HbA1c levels showed no significant improvement after six months of treatment. Interestingly, by 12 months, there was a significant decline in HbA1c (from 9.69% to 8.31%, p=0.001), reflecting improved metabolic control over time. These results are consistent with dulaglutide’s mechanism of enhancing glucose-dependent insulin secretion and suppressing glucagon release [19]. At six months, HbA1c increased slightly to 10.05±11.44%, a change that was not statistically significant (p=0.722). The unusually high standard deviation observed at this time point may reflect inconsistencies in retrospective documentation or variations in clinical follow-up. Therefore, caution is warranted when interpreting the intermediate glycemic outcomes.

From a safety perspective, dulaglutide was well tolerated. In this study, 11% of patients experienced gastrointestinal (GI) side effects, including constipation (2%), nausea (2%), vomiting (3%), diarrhea (1%), and abdominal pain (3%). A smaller proportion (1%) reported a decrease in appetite. These rates are notably lower than those reported in randomized controlled trials (RCTs), where GI adverse events have been observed in up to 30-40% of patients, particularly during the initial weeks of therapy [20]. This suggests a more favorable tolerability profile in our real-world population, possibly due to gradual dose titration or differences in patient characteristics. Notably, 24% of patients experienced hypoglycemia, likely due to the concurrent use of insulin or sulfonylureas, which are known to increase the risk of low blood glucose when combined with GLP-1 receptor agonists [21]. This underscores the importance of individualized medication management and close patient monitoring when prescribing dulaglutide alongside other antidiabetic agents. Meanwhile, 47% of patients reported no adverse effects, and dulaglutide was well tolerated by the majority of participants, supporting its continued use as part of long-term type 2 diabetes management.

This study provides valuable insights into the real-world application of dulaglutide in the Saudi population, where obesity and T2DM represent significant health burdens [5]. However, limitations, such as the retrospective design and the absence of randomization, must be acknowledged. Although lifestyle data were available for the majority of participants, the lack of detailed information on patients lifestyle habits, including dietary intake and physical activity, these variables were either inconsistently documented or entirely unavailable in the medical records, this limits the interpretation of weight outcomes, as unmeasured diet or exercise behaviors could have influenced the results, lifestyle information was missing for 4% of the sample, we did not perform a sensitivity analysis excluding these participants. Although efforts were made to account for key confounding factors, such as age, BMI, and comorbidities, through stratified analyses, the retrospective nature of the study limited our ability to fully adjust for all potential confounders. However, future studies should consider more comprehensive lifestyle data collection and subgroup analyses to isolate pharmacologic effects more clearly, and account for potential confounding factors and more robustly assess long-term outcomes, especially when comparing different GLP-1 receptor agonists.

## Conclusions

Dulaglutide demonstrated a modest short-term weight loss and meaningful glycemic improvement in diabetic Saudi patients, although long-term effects on weight appear limited in our study. The treatment was generally safe and well tolerated, with a low incidence of adverse effects. These findings support the utility of dulaglutide not only for glycemic control but also as a supplementary tool for weight management in patients with T2DM, particularly in populations with high obesity prevalence, such as Saudi Arabia. Future research should focus on combining dulaglutide with lifestyle interventions and comparing its long-term effectiveness with other GLP-1 receptor agonists.

## References

[1] Panuganti KK, Nguyen M, Kshirsagar RK (2025). Obesity. StatPearls [Internet].

[2] (2025). Obesity and overweight. 2024.

[3] Abdelaal M, le Roux CW, Docherty NG (2017). Morbidity and mortality associated with obesity. Ann Transl Med.

[4] Kelly T, Yang W, Chen CS, Reynolds K, He J (2008). Global burden of obesity in 2005 and projections to 2030. Int J Obes (Lond).

[5] Althumiri NA, Basyouni MH, AlMousa N (2021). Obesity in Saudi Arabia in 2020: prevalence, distribution, and its current association with various health conditions. Healthcare (Basel).

[6] Powell-Wiley TM, Poirier P, Burke LE (2021). Obesity and cardiovascular disease: a scientific statement from the American Heart Association. Circulation.

[7] Demeulemeester F, de Punder K, van Heijningen M, van Doesburg F (2021). Obesity as a risk factor for severe COVID-19 and complications: a review. Cells.

[8] Yumuk V, Tsigos C, Fried M, Schindler K, Busetto L, Micic D, Toplak H (2015). European Guidelines for Obesity Management in Adults. Obes Facts.

[9] Collins L, Costello RA (2025). Glucagon-like peptide-1 receptor agonists. StatPearls [Internet].

[10] Glucagon-like peptide-1 (GLP-1) agonist - Incretin mimetics. GlobalRPH. https://globalrph.com/drugs/diabetes-incretin-mimetics/. Accessed July 10 (2025). Glucagon-like peptide-1 (GLP-1) agonist - incretin mimetics. https://globalrph.com/drugs/diabetes-incretin-mimetics/.

[11] Htike ZZ, Zaccardi F, Papamargaritis D, Webb DR, Khunti K, Davies MJ (2017). Efficacy and safety of glucagon-like peptide-1 receptor agonists in type 2 diabetes: a systematic review and mixed-treatment comparison analysis. Diabetes Obes Metab.

[12] Wilding JP, Batterham RL, Calanna S (2021). Once-weekly semaglutide in adults with overweight or obesity. N Engl J Med.

[13] Lee MM, Sattar N, Pop-Busui R (2025). Cardiovascular and kidney outcomes and mortality with long-acting injectable and oral glucagon-like peptide 1 receptor agonists in individuals with type 2 diabetes: a systematic review and meta-analysis of randomized trials. Diabetes Care.

[14] Madsbad S (2014). The role of glucagon-like peptide-1 impairment in obesity and potential therapeutic implications. Diabetes Obes Metab.

[15] Fala L (2015). Trulicity (dulaglutide): a new GLP-1 receptor agonist once-weekly subcutaneous injection approved for the treatment of patients with type 2 diabetes. Am Health Drug Benefits.

[16] Davis TM, Badshah I, Chubb SA, Davis WA (2016). Dose-response relationship between statin therapy and glycaemia in community-based patients with type 2 diabetes: the Fremantle Diabetes Study. Diabetes Obes Metab.

[17] Drucker DJ (2018). Mechanisms of action and therapeutic application of glucagon-like peptide-1. Cell Metab.

[18] Jendle J, Grunberger G, Blevins T, Giorgino F, Hietpas RT, Botros FT (2016). Efficacy and safety of dulaglutide in the treatment of type 2 diabetes: a comprehensive review of the dulaglutide clinical data focusing on the AWARD phase 3 clinical trial program. Diabetes Metab Res Rev.

[19] Gerstein HC, Colhoun HM, Dagenais GR (2019). Dulaglutide and renal outcomes in type 2 diabetes: an exploratory analysis of the REWIND randomised, placebo-controlled trial. Lancet.

[20] Filippatos TD, Panagiotopoulou TV, Elisaf MS (2014). Adverse effects of GLP-1 receptor agonists. Rev Diabet Stud.

[21] ElSayed NA, Aleppo G, Aroda VR (2023). Improving care and promoting health in populations: standards of care in diabetes - 2023. Diabetes Care.

